# The estrogenicity of methylparaben and ethylparaben at doses close to the acceptable daily intake in immature Sprague-Dawley rats

**DOI:** 10.1038/srep25173

**Published:** 2016-04-28

**Authors:** Libei Sun, Tong Yu, Jilong Guo, Zhaobin Zhang, Ying Hu, Xuan Xiao, Yingli Sun, Han Xiao, Junyu Li, Desheng Zhu, Linlin Sai, Jun Li

**Affiliations:** 1 College of Urban and Environmental Sciences, MOE Laboratory for Earth Surface Processes, Peking University, Beijing 100871, China; 2 Laboratory Animal Center, Peking University, Beijing 100871, China; 3 Shandong Academy of Occupational Health and Occupational Medicine, Ji’nan, Shandong 250062, China

## Abstract

The estrogenicity of parabens at human exposure levels has become a focus of concern due to the debate over whether the estrogenicity of parabens is strong enough to play a role in the increased incidence of breast cancer. In this study, the uterotrophic activities of methylparaben (MP) and ethylparaben (EP) at doses close to the acceptable daily intake as allocated by JECFA were demonstrated in immature Sprague-Dawley rats by intragastric administration, and up-regulations of estrogen-responsive biomarker genes were found in uteri of the rats by quantitative real-time RT–PCR (Q-RT-PCR). At the same time, the urinary concentrations of MP and EP, as measured by gas chromatography–mass spectrometry (GC-MS) in rats that received the same doses of MP and EP, were found to be near the high urinary levels reported in human populations in recent years. These results show the *in vivo* estrogenicity of MP and EP at human exposure levels, and indicate that populations exposed to large amounts of MP and EP may have a high burden of estrogenicity-related diseases. In addition, a molecular docking simulation showed interaction between the parabens and the agonist-binding pocket of human estrogen receptor α (hERα).

Parabens are a homologous series of p-hydroxybenzoic acid esters that differ in the ester group. They have been shown to be very effective antimicrobial agents and are used extensively, either individually or in combination, as preservatives in foods, cosmetics, drugs, and toiletries. In 1974, the FAO/WHO Joint Expert Committee on Food Additives (JECFA) allocated a total acceptable daily intake (ADI) of 0 to 10 mg/kg body weight (bw) for the sum of methylparaben (MP), ethylparaben (EP), and propylparaben (PP)^1^. Over the years, the use of parabens has steadily increased to include more categories of foods, such as processed vegetables, baked goods, fats and oils, seasonings, sugar substitutes, coffee extracts, fruit juices, pickles, sauces, soft drinks, and frozen dairy products at concentrations between 450 and 2000 ppm^2^,^3^. In 2007, the JECFA recommended that PP should be excluded from the paraben ADI for use as a preservative in food due to its adverse effects, leaving only MP and EP^4^; many countries have now adopted this recommendation and allow the use of only MP, EP, and their salts as preservatives in foods.

The wide use of parabens has led to continuous human exposure and absorption. Extensive distribution of parabens in human samples, including milk, urine, semen, and blood, has been intensively reported over the past decade^5^,^6^,^7^,^8^,^9^,^10^,^11^,^12^,^13^. An analysis of urinary concentrations of MP, PP, and butylparaben (BP) in the general population in the United States showed that MP, PP, and BP were detectable in 99.9, 98.3, and 73.6% of women and 99.3, 90.2, and 35.9% of men, respectively^12^. In Spain, MP, EP, PP, and BP were measurable in 100, 87.6, 98.3, and 90.1% of urine samples from pregnant women and 100, 80, 100, and 83.3% of urine samples from children, respectively^13^. Similarly, MP, EP, PP, and BP were measurable in 98, 80, 98, and 83% of urine samples from men in Denmark, respectively^8^. A recent analysis of breast tissue samples collected during mastectomy performed for primary breast cancer in England showed that at least one intact paraben ester was detectable in virtually all samples (99%), with a total median value of 85.5 ng/g (range, 0 to 5134.5 ng/g) of tissue for all five parabens^10^; these findings aroused concern over the estrogenicity of parabens at levels of human exposure, because estrogen is known to play a central role in the development, growth, and progression of breast cancer^14^.

The estrogenic properties of parabens have been a focus of concern since the 1990s^3^,^15^, although they have also been shown to have multiple endocrine-disrupting properties such as anti-androgenic activity and inverse antagonist activity on estrogen-related receptor γ^16^,^17^,^18^. In a yeast-based estrogen assay, Routledge *et al.* found that all parabens were weakly estrogenic with the most potent being 10,000-fold less potent than 17β-estradiol (E_2_)^19^. Okubo *et al.* studied the estrogenicity of parabens by evaluating the estrogen receptor–dependent proliferation of MCF-7 cells and found that all of the parabens could stimulate proliferation to about the same level as the maximal cell yield attained with 3 × 10^−11^ M E_2_, but at a concentration on the order of 10^5^ to 10^7^ times higher than E_2_^20^. However, some studies later found that the *in vivo* estrogenicity of parabens was not as low as demonstrated by the *in vitro* assays. Lemini *et al.* studied the estrogenicity of parabens with uterotrophic assay in immature and adult ovariectomized CD1 mice and in immature female Wistar rats^21^. They found the median effective dose (ED_50_) of parabens to increase the uterine weight in CD1 mice ranged from 18 to 74 mg/kg, and that of E_2_ was 7 μg/kg; in Wistar rats, the ED_50_ ranged from 33 to 338 mg/kg^21^. These ED_50_ values are only slightly larger than the ADI value allocated by the JECFA^1^. In a review of studies on paraben toxicology, Darbre and Harvey^15^ discussed whether “parabens [should] be termed ‘weak oestrogens’” and suggested that “the extent to which parabens can be labelled as ‘weak oestrogens’ needs further consideration”^15^.

Because MP and EP are still used as preservatives in foods according to the ADI of 0 to 10 mg/kg bw allocated in 1974^1^, we used uterotrophic assay to investigate the *in vivo* estrogenic effects of MP and EP administered by oral gavage at doses close to the ADI in Sprague-Dawley (SD) rats, which have been proven to be sensitive to estrogen in uterotrophic assay^22^, and analyzed the transcriptional changes of estrogen-responsive genes in uteri and the MP or EP concentrations in rats’ urine.

## Results

### Uterotrophic activities in SD rats

**Figure 1:**
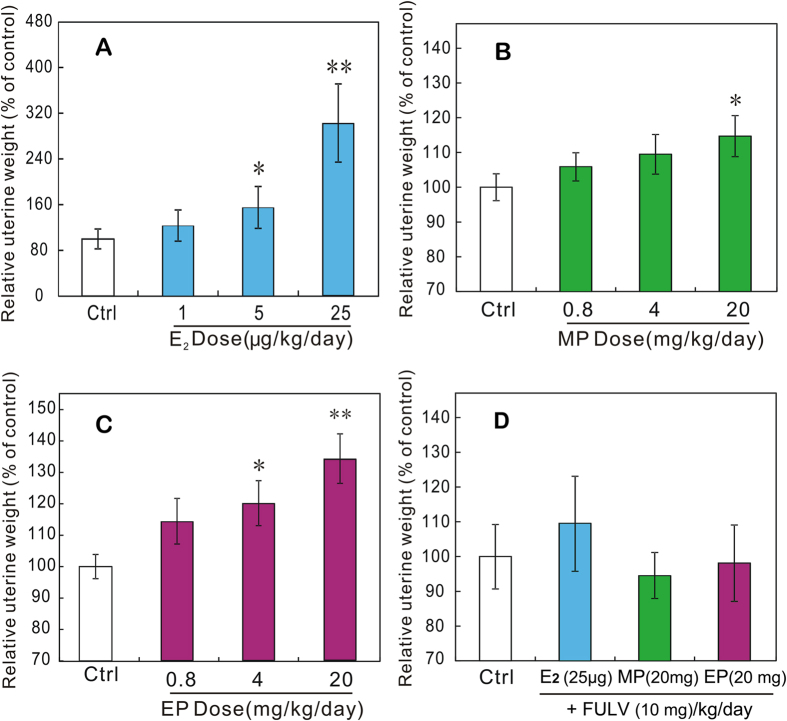
Relative uterine weights of rats that received oral administration of E_2_ (**A**), MP (**B**), or EP (**C**) alone and in combination with FULV (**D**) for 3 days beginning on postnatal day 21. Values are expressed as means ± SDs. Dunnett’s test with corresponding control as reference group was used to estimate uterotrophic effect. *Significantly different from corresponding control (*P* < 0.05). **Significantly different from corresponding control (*P* < 0.01).

The experimental design and the numbers of animals in each groups are listed in Table 1. During the 3 days of treatment, E_2_ elicited significant uterotrophic activity in a dose-related manner; the relative uterine weights were significantly increased in rats that were given 5 and 25 μg/kg bw/day of E_2_ (Fig. 1A). As shown in Fig. 1B,C, the relative uterine weights of rats that were given MP and EP were increased in a dose-dependent manner. The uterine weights were significantly increased in rats treated with 20 mg/kg bw/day of MP and 4 and 20 mg/kg bw/day of EP (P < 0.05); therefore, based on the uterotrophic activities of MP and EP, the lowest-observed-effect levels (LOELs) were 20 mg/kg bw/day for MP and 4 mg/kg bw/day for EP, and the no-observed-effect levels were 4 mg/kg bw/day for MP and 0.8 mg/kg bw/day for EP. The uterotrophic effects were antagonized in rats treated with 25 μg/kg bw/day E_2_, 20 mg/kg bw/day MP, or 20 mg/kg bw/day EP in combination with 10 mg/kg bw/day fulvestrant (FULV) (Fig. 1D).

### Up-regulations of estrogen-responsive genes in uteri of SD rats

**Figure 2:**
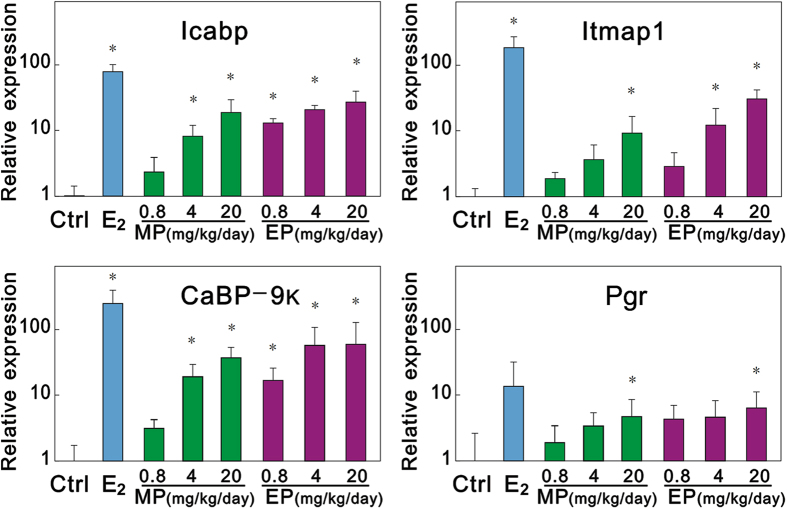
Relative gene expressions in uteri of rats treated with E_2_, MP, or EP alone for 3 days beginning on postnatal day 21. Values are expressed as means ± SDs. Dunnett’s test with corresponding control as reference group was used to estimate gene expression changes. *Significantly different from corresponding control (vehicle control; *P* < 0.05).

**Figure 3:**
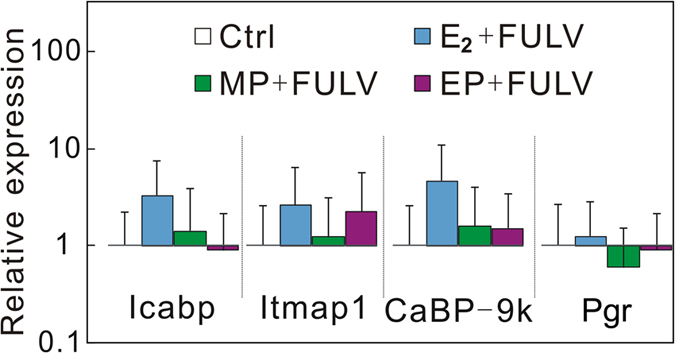
Relative gene expressions in uteri of rats treated with E_2_, MP, or EP in combination with FULV for 3 days beginning on postnatal day 21. Values are expressed as means ± SDs.

To verify that the uterotrophic activity was caused by *in vivo* estrogenicity, the relative expressions of estrogen-responsive genes in the uteri of SD rats treated with E_2_, MP, and EP alone or in combination with antiestrogen FULV were analyzed by means of quantitative real-time RT-PCR (Q-RT-PCR) (Table 2). Among the seven candidate estrogen-responsive genes, intestinal calcium-binding protein (icabp), integral membrane-associated protein-1 (itmap1), calbindin-D 9k (CaBP-9k), and progesterone receptor (Pgr) were found to be highly up-regulated by 25 μg/kg bw/day E_2_; these values reached 79-fold, 186-fold, 254-fold, and 13.6-fold of control values, respectively. We then selected icabp, itmap1, CaBP-9k, and Pgr as estrogen-responsive biomarker genes and analyzed their transcriptional levels. As shown in Fig. 2, the relative expression levels of icabp, itmap1, CaBP-9k, and Pgr were increased in a dose-dependent manner after administration of MP or EP alone. The expression of icabp and CaBP-9k was significantly up-regulated by 4 and 20 mg/kg bw/day of MP and all doses of EP. Itmap1 was significantly up-regulated by 20 mg/kg bw/day of MP and 4 and 20 mg/kg bw/day of EP. Pgr was significantly up-regulated by 20 mg/kg bw/day of MP and 20 mg/kg bw/day of EP. In rats treated with 25 μg/kg bw/day E_2_, 20 mg/kg bw/day MP, or 20 mg/kg bw/day EP in combination with 10 mg/kg bw/day FULV, the expression of these estrogen-responsive genes returned to levels similar to that of the control (Fig. 3).

### Levels of MP and EP in urine of SD rats

We determined the concentrations of intact parabens (free plus conjugated) of MP and EP in the urine of rats after oral administration of MP and EP (Table 3). The urinary concentrations of the MP and EP were both increased in a dose-dependent manner, which ranged from 491 ± 73.0 ng/ml to 17,635 ± 5592 ng/ml for MP and from 376 ± 67.1 ng/ml to 11,906 ± 5584 ng/ml for EP in rats that received oral doses from 0.8 to 20 mg/kg/day. The amount of the intact parabens (free plus conjugated) recovered in the urine ranged from 5.10% ± 0.76% to 7.19% ± 2.18% for MP and from 3.92% ± 0.84% to 4.79% ± 2.18% for EP.

### Interaction of human estrogen receptor α ligand-binding domain with MP or EP

**Figure 4:**
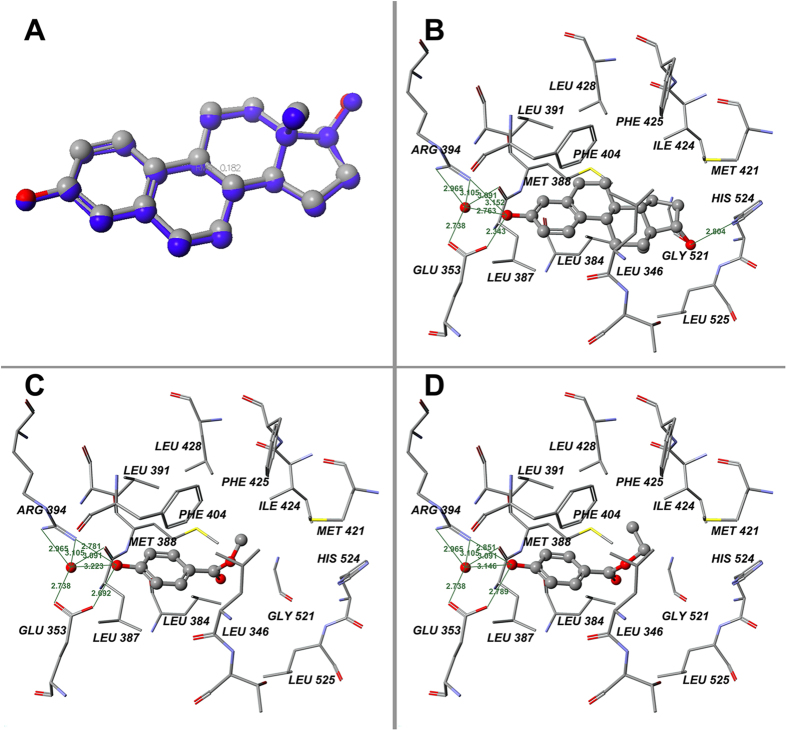
Results of docking calculations on hERα-LBD. (**A**) Validation of docking of 1ERE with E_2_: docked ligand (blue) and ligand of crystal structure at their absolute positions in binding pocket; (**B**) Binding positions of original ligand E_2_ on hERα-LBD in x-ray structure (1ERE chain A) template; (**C**) Binding positions of MP; (**D**) Binding positions of EP. Green lines indicate hydrogen bonds between chemicals and amino acid residues. Green numerals indicate distances between two atoms (Å).

To better understand the estrogenicity, MP and EP were docked into the agonist pocket of human estrogen receptor α (hERα) ligand-binding domain (LBD). For validation of the method’s reliability, the ligand E_2_ was docked into the agonist pocket of hERα-LBD. As is shown in Fig. 4A, the root-mean-square error between the original and the calculated E_2_ was 0.182 Å, which indicates the reliability of this *in silico* method. The interaction between E_2_ and the receptor is shown in Fig. 4B, and the binding poses of MP and EP in the agonist pocket of hERα-LBD are shown in Fig. 4C,D. The binding energies of MP and EP were calculated to be −49.35 and −53.38 kcal/mol, respectively. The important feature of the interaction between the parabens and the ligand-binding pocket of hERα was the formation of hydrogen bonds between the p-hydroxyl group of parabens and the Glu353/Arg394 of the pocket, either directly or via mediation of a single molecule of water in addition to the hydrophobic interactions between the phenyl ring and alkyl group of parabens and the core hydrophobic moiety provided by PHE404, PHE425, LEU346, LEU384, LEU428, LEU391, LEU525, MET388, MET421, and ILE424.

## Discussion

This study demonstrates the *in vivo* estrogenicity of MP and EP at doses close to the ADI in immature SD rats after intragastric administration. The subpopulation of SD rats used in this study has been proven to be sensitive to estrogen by uterotrophic assay and was used to evaluate benzylparaben in a recent study by our group^22^. As shown in Fig. 1, both MP and EP significantly increased the uterine weight in rats after 3 days of exposure. The no-observed-effect levels of MP and EP were 4 mg/kg bw/day and 0.8 mg/kg bw/day, respectively, which were similar to the no-observed-effect levels (5.5 mg/kg bw/day of MP and 0.6 mg/kg bw/day of EP) reported in the immature CD-1 mice uterotrophic assay by subcutaneous injection^21^. Because uterotrophic activity usually reflects the *in vivo* estrogenicity of a chemical and because the uterotrophic effects induced by MP and EP could be antagonized by FULV, which is an estrogen-receptor antagonist with no uterotrophic effects on immature rats and blocks the agonistic effects of E_2_ in a dose-dependent manner^23^, the uterotrophic activities of MP and EP indicate their estrogenicity *in vivo*. In fact, some of the new findings also reveal the *in vitro* estrogenicity of parabens at low levels. Charles and Darbre^24^ showed that the LOELs of MP and EP for the proliferation of MCF-7 cells after 7 days of exposure were 6 × 10^−5^ M and 2 × 10^−6^ M, respectively^24^. These results indicated that the ADI of parabens allocated by the JECFA may be too high to protect people’s health.

By measuring gene expression, four estrogen-responsive biomarker genes (icabp, itmap1, CaBP-9k, and Pgr) were found to be highly up-regulated in the uteri of the rats treated with doses of MP and EP, which validates the *in vivo* estrogenicity of MP and EP. Icabp, itmap1, and Pgr have been reported to be up-regulated in the uteri and ovaries of rats treated with xenoestrogens (17 α-ethynyl estradiol, bisphenol A, and genistein)^25^,^26^. The CaBP-9k gene is an estrogen-responsive biomarker gene in rat uteri. The expression of the CaBP-9k gene is up-regulated by estrogen and down-regulated by P4 during the estrous cycle and during early pregnancy in the rat uterus^27^,^28^. Vo and Jeung^29^ reported that PP, BP, isopropylparaben, and isobutylparaben could up-regulate the gene expression of CaBP-9k in rat uteri^29^. This study demonstrates that MP and EP can also up-regulate the gene expression of CaBP-9k in rat uteri (Fig. 2). Vo *et al.* reported that CaBP-9k mRNA and protein expression can be significantly induced by MP, EP, and other parabens at a level of 10^−4^ M (approximately 15.2 ppm for MP and 16.6 ppm for EP, which are near the doses of MP and EP used in this study) in GH3 rat pituitary cancer cells and demonstrated that estrogen receptor is involved in the induction of CaBP-9k by parabens in GH3 cells^30^. The up-regulation of icabp, itmap1, CaBP-9k, and Pgr observed in this study provides evidence to support the *in vivo* estrogenicity of MP and EP. The *in silico* molecular docking experiment in our study revealed that the charged/polarized residues, Glu353 and Arg394, that existed at the ligand-binding site of the estrogen receptor played a key role in estrogen receptor–paraben binding; they constructed hydrogen bonds with the ligand, directly or via mediation of a single molecule of water (Fig. 3). The Glu353 and Arg394 of hERα are also known to play a critical role in hERα–E_2_ binding^31^. In addition to estrogen receptors, it is known that parabens can act as antagonists of androgen receptors and inhibitors of sulfotransferase enzymes^32^,^33^,^34^. Our recent study demonstrated that parabens possessed obvious agonist/inverse antagonist activities on ERRγ at very low levels (LOEL of 10^−7^ M)^18^; this receptor was reported to play an obligatory role in the induction of cytochrome P450 aromatase (CYP19) expression in human trophoblasts, and aromatase is known to be an estrogen synthetase that is responsible for a key step in the biosynthesis of estrogens^35^. All of these biological activities may contribute to the *in vivo* estrogenicity of MP and EP.

Parabens and their metabolites, alkyl protocatechuates, are known as biomarkers for paraben exposure^7^,^36^. Because more data on the urinary concentrations of parabens have been reported, this study used urinary parabens (free plus conjugated) as biomarkers for comparison with the concentrations reported in human urine samples. Among parabens, MP is the most commonly used paraben and is detectable in almost all human uterine samples. Among patients from the Fertility Center at the Massachusetts General Hospital, MP was detected in 99.9% of women and 99.3% of men, with maximal urinary MP concentrations of 15,100 μg/L in women and 23,200 μg/L in men^12^. EP is usually used less than MP in amount and frequency, of which the median concentration in urine from young Danish men was reported to be 1.98 ng/ml with a maximum of 564 ng/ml^8^. The urinary MP concentration (17,635 ± 5592 ng/ml) of rats treated with 20 mg/kg bw/day (the LOEL observed in the uterotrophic assay of this study) of MP and the urinary EP concentration (2051 ± 1098 ng/ml) in rats treated with 4 mg/kg bw/day (the LOEL) of EP are close to the highest human urine levels in human populations. In addition, it is notable that increasing amounts of MP and EP may be used in the future due to the removal of PP from the paraben ADI.

In conclusion, this study demonstrates the uterotrophic activities of MP and EP at doses near the ADI allocated by the JECFA by intragastric administration in immature SD rats and demonstrates up-regulation of estrogen-responsive biomarker genes in the rats’ uteri, indicating the *in vivo* estrogenicity of MP and EP at levels of human exposure. At the same time, the concentrations of MP and EP in urine from rats treated with the same doses of MP and EP, in which *in vivo* estrogenic effects were observed, were close to the highest urinary levels reported in human populations in recent years, which suggests that the populations, who are usually exposed to large amounts of MP and EP, may have a high burden of diseases related to estrogenicity.

## Materials and Methods

### Chemicals

MeOH and hexane were HPLC grade obtained from Fisher Chemicals (Fair Lawn, NJ). E_2_ (>98.0%) was purchased from Sigma-Aldrich (St. Louis, MO), and MP (>99.0%) and EP (>99.5%) were obtained from Hengye Zhongyuan Chemical Co. (Beijing, China)^13^.C_6_-MP were purchased from Cambridge Isotope Laboratories (Andover, MA). All chemicals and reagents used in this study were of molecular biology grade unless otherwise specified.

### Animals and Experimental Design

We carried out four rounds of experiments with oral gavage doses of compounds in immature female SD rats at 20 postnatal days of age (Table 1). The rats were purchased from the Experimental Animal Tech Co. of Weitonglihua (Beijing, China). The weight variation in the animals used was less than 20% of the mean weight. The rats were housed in twos or threes in stainless steel wire-mesh cages. The housing environment was controlled at a temperature of 22 °C ± 2 °C, a relative humidity of 40–60%, and a 12-h/12-h light/dark cycle. The rats were fed a basic diet from the Laboratory Animal Center of the Academy of Military Medical Sciences (Beijing, China) *ad libitum* and had access to sufficient drinking water. Before the experiment, the rats were randomly assigned to treatment and control groups. The numbers of animals in each group are listed in Table 1. The body weights were measured and recorded daily throughout the experiments. Intragastric administration of the control and test compounds to each rat was performed daily for 3 days beginning on postnatal day 21 according to the weight of the rat. The tested chemicals were dissolved in peanut oil for intragastric administration (5 mL/kg bw). The control group was treated with peanut oil only for each of the experiments. In experiment 1, E_2_ was prepared at doses of 1, 5, and 25 μg/kg bw/day; the rats were weighed and sacrificed 24 h after the final treatment. Each uterus was dissected and weighed to study the uterotrophic effect; the uterus was frozen in liquid nitrogen until RNA isolation, and a relative uterine weight was calculated for each animal according to the following equation:


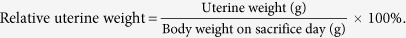
 Dunnett’s test with the control as reference group was used to estimate the uterotrophic effect.

In experiment 2, MP and EP were prepared at doses of 0.8, 4, and 20 mg/kg bw/day, and the uterotrophic effects of MP and EP were determined with the same experimental protocol as in experiment 1. In experiment 3, doses of 25 μg/kg bw/day E_2_, 20 mg/kg bw/day MP, or 20 mg/kg bw/day EP combined with 10 mg/kg bw/day FULV were prepared, and the uterotrophic effects were studied using the same experimental protocol as in experiment 1. Experiment 4 included the same doses of MP and EP as in experiment 2; after the final treatment on postnatal day 23, the rats were moved from their experimental cages to metabolic cages in which each rat was individually housed for 24 hours with only pure water ad libitum. The metabolic cages were designed to prevent fecal contamination of urine. The urine was collected in polypropylene tubes and stored at −80 °C for MP and EP analysis. All animal studies were approved by the Institutional Animal Care and Use Committee of Peking University and were performed in accordance with the Guidelines for Animal Experiments of the university, which meet the ethical guidelines for experimental animals in China.

### Quantitative real-time RT-PCR assay

Total RNA was isolated from each uterus with 1 ml of Trizol reagent (Gibco BRL, Life Technologies, Gaithersburg, MA), according to the manufacturer’s instructions. The total RNA was digested by DNase I (TaKaRa Biotechnology, Dalian, China) to remove genomic DNA contamination. The purified total RNA was measured at 260 and 280 nm using a NanoDrop spectrophotometer. The 260:280-nm ratio and 1% agarose-formaldehyde gel stained with ethidium bromide were used to verify the quality of the RNA in each sample. Six samples from each group were used for Q-RT-PCR assay. Synthesis of first-strand cDNA was performed using AMV Reverse Transcriptase and other reverse transcription reagents from Promega Corporation (Madison, WI). Primers for quantification of mRNA of each gene were designed using Primer Express 3.0 (Applied Biosystems, Foster City, CA) and validated by Premier Primer 5.0 (Premier Biosoft International, Palo Alto, CA) and are shown in Table 2. The pairs of primers were designed to span at least one intron of the genomic sequence. Quantitative real-time PCR with SYBR green detection was performed with an Agilent Mx3005P QPCR System. The PCR reaction mixture contained 12.5 μL of 2 × SYBR Green real-time PCR master mix (Toyobo, Shanghai), 250 nM each of forward and reverse primer, 1 μL of cDNA template, and nuclease-free water at a total volume of 25 μL. The reactions were incubated at 95 °C for 1 min, followed by 40 cycles of 95 °C for 15 s and 60 °C for 60 s. After the final cycle of PCR, the reactions were denatured over a 35 °C temperature gradient at 0.03 °C/s from 60 °C to 95 °C to determine the quality of the PCR products. Beta-actin (actb) and glyceraldehyde 3-phosphate dehydrogenase (gapdh) were used as the endogenous controls, and relative expression was evaluated by the methods provided by Applied Biosystems. Dunnett’s test with the corresponding control as reference group was used to estimate the gene expression changes. A P-value of less than 0.05 was considered to indicate statistical significance.

### Analysis of MP and EP in rat urine by gas chromatography–mass spectrometry

In this study, intact parabens (free plus conjugated) were analyzed in the rat urine samples from experiment 4 by gas chromatography–mass spectrometry (GC-MS). The details of the analytical methods mostly followed those of previous studies^37^,^38^,^39^. Briefly, 1 mL of each urine sample was transferred into a 15-mL polypropylene tube, spiked with 20 ng^13^C_6_-MP, and buffered with 200 μL of 1 M ammonium acetate. Then, 0.5 mL Milli-Q water was added and the pH was adjusted to 5.5 with ammonium acetate. For deconjugation, 50 μL β-glucuronidase/arylsulfatase (Sigma-Aldrich) was added to the sample and incubated at 37 °C for 20 h. The samples were then diluted and loaded onto a phenomenex strata-x SPE cartridge (200 mg/3 mL) that was previously conditioned with 6 mL MeOH and 3 mL distilled water at a flow rate of 1 mL/min. The cartridge was washed with 3 mL distilled water and 4 mL MeOH/water (1:20, v/v) and dried under a flow of nitrogen. A volume of 4 mL MeOH was used to elute the samples. The eluate was evaporated to dryness and derived by 100 μL BSTFA + 1% TMCS (Regis Technologies, Inc., Morton Grove, IL). After 30 min, the derivatization reaction was stopped by adding 1 mL of water, and the derived sample was extracted with n-hexane (1:1) three times. The extraction was evaporated to dryness under a gentle nitrogen stream and finally dissolved with 100 μL of n-hexane for GC-MS analysis with an Agilent 7890A/5975C GC-MS system. An HP-5MS capillary column (30 m × 0.25 mm × 0.25 μm film thickness; Agilent) was used to separate the target chemical. The details of the instrumental analysis method mostly followed those of previous studies^37^. The mass selective detector was operated in selected ion monitoring mode; mass ions used for MP were 209, 224, and 135; and those used for EP were 223, 193, and 238. The mean recoveries (%), precision (% RSD), and LOD with coefficients (R2) > 0.996 were 91.6 ± 5.66, 5.79, and 0.68 ng/ml for MP and 94.6 ± 4.28, 4.87, and 0.89 ng/ml for EP, respectively. The recoveries of parabens (free plus conjugated) in urine were calculated according to the following equation:


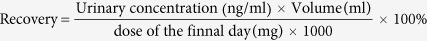


### Automated molecular docking

Scigress (Version 3.0; Fujitsu) was used to perform the *in silico* molecular docking studies. The chemical structures of the test chemicals and original ligands were drawn, cleaned, and energy optimized with procedures integrated in Scigress. The crystallographic three-dimensional structure of the hERα-LBD (PDB ID 1ERE) was downloaded from the Protein Data Bank website (http://www.rcsb.org/pdb). The structures were refined and reduced to a monomer of chain A, and all water molecules were removed from the protein except for HOH3, which is important to the composition of the ligand-binding pocket. Hydrogen atoms of the target protein, together with the original ligand and water molecules, were subsequently added through the Workspace module of Scigress. Docking calculations were evaluated with a 15 × 15 × 15 Å grid box with 0.25-Å grid spacing. The procedure was set to run 30,000 generations with an initial population size of 80, an elitism of 8, a crossover rate of 0.8, and a mutation rate of 0.2. The potential of mean force, a knowledge-based approach that extracts pairwise atomic potentials from the structural information of known protein-ligand complexes contained in the Protein Data Bank, was used to evaluate the binding affinity of a chemical in the active site. Docking of E_2_, the original ligand of the crystallographic structure, into the agonist pocket of hERα-LBD was performed to validate the reliability of the method.

### Statistical analysis

The statistical program SPSS (version 13.0, Chicago, IL) was used to analyze the data. Group differences were assessed by one-way analysis of variance and Dunnett’s test. A P-value of less than 0.05 was considered to indicate statistical significance. The data are presented as means and standard deviations unless otherwise specified.

## Additional Information

**How to cite this article**: Sun, L. *et al.* The estrogenicity of methylparaben and ethylparaben at doses close to the acceptable daily intake in immature Sprague-Dawley rats. *Sci. Rep.* **6**, 25173; doi: 10.1038/srep25173 (2016).

## Figures and Tables

**Table 1:** Experimental design and number of rats used during experiments.

	Chemicals	Doses (mg/kg bw/day)	Rats (N)
Experiment 1	Control^a^	0	9
	E_2_	0.001	8
		0.005	8
		0.025	8
Experiment 2	Control^a^	0	17
	MP	0.8	8
		4	8
		20	8
	EP	0.8	8
		4	8
		20	8
Experiment 3	Control^a^	0	6
	E_2_+FULV MP+FULV	10 + 0.025	6
		10 + 20	6
	EP+FULV	10 + 20	6
Experiment 4	Control^a^	0	6
	MP	0.8	6
		4	6
		20	6
	EP	0.8	6
		4	6
		20	6

^a^Controls received peanut oil vehicle (5 mL/kg bw) only.

**Table 2:** List of primers for amplifying estrogen-responsive genes in rat uteri.

Gene symbol	Gene full name	Sequences (up, forward primers; down, reverse primers; from 5′ to 3′)
Actb	β-actin	GTCGTACCACTGGCATTGTG
		CTCTCAGCTGTGGTGGTGAA
Hsd11b2	11-beta-hydroxylsteroid dehydrogenase type 2	CCTCCAAGGCAGCTATTGCA
		TCACTGCCTCTGTCTTGAAGCA
CaBP-9k	calbindin-D	AAGAGCATTTTTCAAAAATA
		GTCTCAGAATTTGCTTTATT
Pgr	progesterone receptor	GATGGAAGGGCAGCATAACTATTT
		ACAGCACTTTCTCAGACGACATG
Icabp	intestinal calcium-binding protein	CTGGATAAGAACGATGATGGAGAA
		GGTGGTGTCGGAGCTCCTT
Itmap1	integral membrane- associated protein-1	CTATTTCTTTTCCTCTGGTACCACTATTC
		AGGGTGTGGCCTTGGATAATT
Perl	period 1	CTGCAGGTTCAGGCCTCAAG
		GTTAGGCGGAATGGCTGGTA
EET-1	estrogen-enhanced transcript-1	GCTGTCCTTCCTGCAACAAGAT
		ACGCATCCCAGCAGACACA
Gapdh	Glyceraldehyde 3-phosphate dehydrogenase	CTACCCACGGCAAGTTCAAC
		CCAGTAGACTCCACGACATAC

**Table 3:** Concentrations and recoveries of MP and EP in urine of rats that received oral doses of MP and EP.

Chemicals	Doses (mg/kg/day)	Urinary concentration (ng/mL)	Recovery (%)^a^
Control	0	MP (2.75 ± 2.95)/EP (ND)	–
MP	0.8	491 ± 73.0	5.10 ± 0.76
	4	2750 ± 89.1	5.57 ± 0.48
	20	17,635 ± 5592	7.19 ± 2.18
EP	0.8	376 ± 67.1	3.92 ± 0.84
	4	2051 ± 1098	4.10 ± 2.20
	20	11,906 ± 5584	4.79 ± 2.18

^a^Percentage of doses recovered in urine.
